# Extracts from Salvia-Nelumbinis naturalis alleviate hepatosteatosis via improving hepatic insulin sensitivity

**DOI:** 10.1186/s12967-014-0236-8

**Published:** 2014-08-27

**Authors:** Li Zhang, Jiaoya Xu, Haiyan Song, Zemin Yao, Guang Ji

**Affiliations:** Institute of Digestive Disease, Longhua Hospital, Shanghai University of Traditional Chinese Medicine, Shanghai, China; Department of Biochemistry, Microbiology & Immunology, Ottawa Institute of Systems Biology, University of Ottawa, Ottawa, Canada; E-Institute of Shanghai Municipal Education Commission, Shanghai University of Traditional Chinese Medicine, Shanghai, China

**Keywords:** Hepatosteatosis, Insulin resistance, Salvia-Nelumbinis naturalis

## Abstract

**Background:**

Salvia-Nelumbinis naturalis (SNN), initially called Jiangzhi Granula as a formulae of Chinese medicinal decoction, has been used clinically to treat non-alcoholic fatty liver disease (NAFLD) and related syndromes. The mechanism of SNN action is unknown.

**Methods:**

HepG2 cells were cultured in lipid-rich media supplemented with chemical components of SNN. Male Wistar rats (6 weeks of age) were fed a high calorie diet (15% fat, 15% sucrose, and 2% cholesterol) for eight weeks, and then treated with SNN for four weeks. Body and liver weight, lipids profiles, insulin and glucose levels, glucose and insulin tolerance were evaluated, the mRNA and protein expression of insulin receptor (InsR), insulin receptor substrate (IRS) 1/2, protein kinase B (PKB/Akt), protein expression of suppressor of cytokine signaling 3 (SOCS3), protein kinase C epsilon (PKC ε) in liver tissue were analysed.

**Results:**

Treatment with SNN components in lipid-laden HepG2 cells decreased lipid accumulation. Rats fed with a HC diet developed hepatosteatosis and accompanied hyperglycemia, hyperinsulinemia, hyperleptinemia, and diabetic dyslipidemia. Prolonged HC diet feeding resulted in parabolic response in plasma triglyceride (TG) concentrations, indicative of compromised hepatic production of TG-rich lipoproteins. HC diet feeding also resulted in impaired insulin sensitivity and hepatic insulin signalling. Administration of SNN extracts alleviated hepatosteatosis and conferred to a normolipoproteinemia profile in the HC diet-fed rats. The efficacy of SNN extract in improving liver function and insulin sensitivity was comparable to that of simvastatin or pioglitazone. The improved insulin signaling by SNN treatment was associated with increased IRS and Akt phosphorylation and decreased SOCS3 expression. However, SNN failed to inhibit the PKC ε expression in the liver.

**Conclusions:**

SNN is effective in reducing lipid accumulation in HepG2 cells and attenuating hepatosteatosis in HC diet-fed rats. Reduced hepatic lipid content in the rat liver was associated with improved insulin signalling.

## Background

Hepatosteatosis, i.e. excess accumulation of triglyceride (TG) in the liver, is the defining feature of non-alcoholic fatty liver disease (NAFLD). As the epidemic of obesity has increased worldwide, NAFLD become a critical public health issue because of its high prevalence, potential progression to severe liver disease, and association with serious metabolic abnormalities including type 2 diabetes mellitus, metabolic syndrome, and coronary heart disease [1]. Previously considered as an abnormality confined to adult populations, NAFLD, is now an alarming health issue among obese children [2]. Hence, finding an effective NAFLD therapy is necessary to alleviate the health burden caused by this disease.

Insulin resistance is intimately associated with the pathogenesis of NAFLD, as evidenced by patho-physiologic studies [3–5]. Insulin binds to its receptor, which results in the tyrosine phosphorylation of insulin receptor substrate (IRS)-1and IRS-2, and subsequently activates downstream molecules such as phosphoinositide 3-kinase, protein kinase B (PKB/Akt), etc. [6]. Impaired insulin signalling, especially the paradox of selective hepatic insulin resistance of failing to suppress glucose production but preserved insulin sensitivity in lipid synthesis aggravates insulin resistance and contributes to the development of NAFLD, while regimens that reverse insulin resistance can ameliorate steatosis [7]. Although numerous studies have focused on NAFLD, suggested treatments (thiazolidinediones, statins) do not always produce satisfied results. Calorie restriction and exercise can treat simple or moderate NAFLD effectively, but target drugs are needed when the disease becomes progressive and severe.

For centuries, natural herbal products derived from systemic Traditional Chinese Medicine (TCM) theory and practice have been used to treat nearly all kinds of ailments in China. Formulae, the form of prescription in TCM, typically consists of several medicinal herbs, of which one represents a principal component, and others serve as an adjuvant in assisting the effects or facilitating the delivery of the principal component. Entirely based on TCM theory and NAFLD pathology, Salvia-Nelumbinis naturalis (SNN) formulae (initially called Jiangzhi Granula) was designed, in which *Salviae*as being the principal element and *Nelumbinis*, *Rhizoma Polygoni Cuspidati*, *Herba Artemisiae Scopariae* the ancillary components. We have shown that administration of SNN formulae, together with behavioral intervention, to patients with NAFLD for 24 weeks gave rise to a higher remission rate as compared to behavioral intervention alone (76.56% versus 52.54%) [8]. In addition, with all the cases observed, SNN did not demonstrate any adverse effect during the whole process [8]. These promising clinical observations of the SNN formulae in ameliorating NAFLD have prompted us to define mechanisms responsible for SNN action. An UPLC-MS method has been established to determine the main active components of SNN [9], thus ensuring standardized application in clinical and preclinical studies.

High calorie (HC) diet is a method that is extensively used to induce hepatic steatosis and insulin resistance in rats and mice [10–12], which resemble characteristics of NAFLD in human. In this study, a rat model of HC-induced NAFLD was used to examine the influence of the extract of SNN on NAFLD and its potential role in improving insulin resistance.

## Methods

### Materials

HepG2 cells were purchased from the American Type Culture Collection (ATCC) bioresource center (Manassas, VA, USA). Dulbecco’s modified Eagle’s medium (DMEM), fetal bovine serum (FBS), and penicillin and streptomycin were from Biowest (Nuaillé, France). The main chemical components of the SNN extract protopanaxadiol, tanshinone IIA, gypenoside, Salvianic acid A sodium, and Salvianolic acid B were obtained from Shanghai Winherb Medical Sci & Tech Development Co. Ltd. (Shanghai, China). Pioglitazone were purchased from Sigma (St. Louis, MO, USA). All other reagents were of analytical grade.

### Cell culture

HepG2 were cultured in DMEM supplemented with 10% heat-inactivated fetal FBS, penicillin at 100 U/mL, and streptomycin at 100 μg/mL at 37°C in a 5% CO2 atmosphere. The experiments, started when the cells grew to 80-90% confluence. Cellular steatosis was induced as previously described [13], in brief, HepG2 cells were seeded on 6-well plates 1× 10^5^ cells/well, stimulated by a mixture of 1 mmol/L FFA (the ratio of oleate to palmitate is 2:1) in DMEM containing 1% BSA for 24 h. The steatotic cells were then treated with the chemical components (at 5, 50, and 500 μmol/L concentration for each component) for another 24 h. The cells were stained with Oil-Red O and imaged.

### Preparation of extract of *Salvia-Nelumbinisnaturalis*

The *Salvia-Nelumbinisnaturalis*(SNN) formula composed of *Salviae* (Danshen in China) as the principal element and *Nelumbinis* (Heye in China)*, Rhizoma Polygoni Cuspidati* (Huzhang in China)*, Herba Artemisiae Scopariae* (Yinchen in China) as ancillary components. The plant specimens were under a micro plant grinding machine, triturated and blended to powder, and the powder was then mixed with water/methanol (5:95, V/V) by sonication (42 kHz, 30 min) using an ultrasonic processor as described previously [9]. The main chemical components of the SNN extract has been determined by UPLC-MS system in previous publications [9].

### Animals and study design

Male Wistar rats were obtained from Slac Animal Laboratories (Shanghai, China), and housed under a standard 12-h light–dark cycle (lights on at 7:00 AM) with access to food and water *ad libitum*. After approximately 1-week acclimation, rats were placed on either standard chow or HC (15% fat, 15% sucrose and 2% cholesterol) diet for up to 12 weeks. During the last 4-week dieting, rats on HC diet were divided into four groups – one group continued on HC diet and the remainder three groups were administered (through oral gavage, daily) with pioglitazone (10 mg/kg body weight), Simvastatin (4 mg/kg), and SNN extract (600 mg/kg), respectively. Oral gavage of saline was applied to control rats. The experimental protocols were approved by Shanghai University of Traditional Chinese Medicine. Rats were housed in a pathogen free environment, and body weight and 24-h food intake were measured weekly.

### Oral glucose tolerance test and insulin tolerance test

Oral glucose tolerance test (OGTT) and insulin tolerance test (ITT)were performed with rats at the end of dieting using previously described methods [14]. Briefly, rats were fasted for 6 h after the start of the light cycle, then orally administered with glucose (1.5 g/kg body weight, for OGTT) or intraperitoneally administrtered with insulin (0.75u/kg body weight, for ITT), tail-vein blood samples were collected at baseline and at indicated time intervals (15, 30, 60, 90, and 120 min) after glucose/insulin administration. Blood glucose levels were determined with a diabetes monitoring strip (Lifescan One Touch, IN). The curve was drawn and the area under the curve (AUC) was calculated.

### Serum biochemical analysis

After fasting for 12 h, rats were anaesthetized with sodium pentobarbital (100 mg/kg) and sacrificed, and blood was collected from aorta abdominalis. Serum TG, total cholesterol (TC), high density lipid-cholesterol (HDL-c), low density lipid-cholesterol (LDL-c), free fatty acids (FFA), fasting blood glucose (FBG), almandine aminotransferase (ALT), and aspartate aminotransferase (AST) were analyzed using the Hitachi full-automatic system. Fasting insulin (FIN) was measured using the standard radio-immunity kits (Puerweiye Bioengineering Institute, Beijing, China).

### Hepatic histology assessment

Liver sections were stained with Hematoxylin and eosin (HE) and Oil-Red O (Sigma, St. Louis, MO), the procedures were performed according to previously describe methods [15]. Briefly, the liver tissues were fixed in 10% neutral buffered formalin for 24 h, dehydrated and embedded in paraffin, the sections were cut, deparaffinized and stained with HE. Snap frozen tissues were placed in optimal cutting temperature compound and then sectioned and stained with Oil-Red O.

### Hepatic lipid and glycogen content

Liver TG was estimated as described previously [16]. Briefly, liver tissue (200 mg) was homogenized in 3 ml of ethanol-acetone (1:1) mixture. The homogenate was extracted over night at 4°C, and centrifuged for 15 min at 3,000 rpm at 4°C. The organic layer was removed, TG and cholesterol were measured using commercial kits (Kangtai Bioengineering Institute, Beijing, China). A standard kit (Jianchen tech, Nanjing, China) was used to determine the hepatic glycogen concentration.

### RNA analysis

Total RNA was extracted from liver and hypothalamic tissues by using a TRIzol Reagent (Invitrogen Corp., Carlsbad, CA) following the manufacturer’s instructions. A reverse-transcription system (Promega Corp., Madison, WI) was then used to transcribe the RNA. Semi-quantitative reverse transcription polymerase chain reaction method was performed using Mastercycler (Eppendorf, Inc., Germany). The primer sequences are shown in Table 1. The mRNA levels of all genes were normalized using β-actin as an internal control.

**Table 1:** **Sequences of the primers used for amplification of mRNA by PCR**

**Gene**	**Forward primer**	**Reverse primer**
InsR	TCTTCAGGGCAATGTCGTTC	GCTCCTATGCTCTGGTGTCA
IRS1	TGCTGGTGGAAGAGGAGG	TGGACAAACGGAGTAGGG
IRS2	GACCAAGTCGGTGAGTGC	GCCCGAACCTCAATAACA
β-actin	CTGGAAGGTGGACAGTGAG	GAGGGAAATCGTGCGTGAC

### Tissue sampling and western blot analyses

Frozen liver and hypothalamic tissues were homogenized in Tissue Protein Extraction Reagent (Pierce Biotechnology, Inc., Rockford, USA), with the addition of protease inhibitor (Roche, Nutley, USA) and phosphatase inhibitor cocktail (Roche, Nutley, USA). Protein concentrations were determined using the bicinchoninic assay reagents and the micro-bicinchoninic assay method (Pierce Biotechnology, Inc. Rockford, USA). For Western blot analysis, 100 μg of protein were fractionated by 8-12% SDS-PAGE, transferred onto a PVDF membrane (Bio-Rad, Hercules, CA), Membranes were blocked with 5% skim milk in Tris-buffered saline and probed with target primary and secondary antibodies. Total and phosphorylated InsR (Tyr^1131^), IRS1 (Ser^302^), Akt (Ser^473^) and SOCS3 antibodies were purchased from Cell Signaling Technology (Danvers, MA, USA), PKCε antibody was from Boster company (Wuhan, China). β-actin and secondary antibodies (from mouse or rabbit) conjugated to peroxidase were purchased from Santa Cruz Biotechnology. The targeted proteins were detected with ECL Detection Kit (Millipore, Billerica, USA), images were taken and qualified by Gel-Pro system (Tanon Technologies).For western blot analysis, the amount of protein loaded was confirmed by the Bradford method, and equal loading was verified by staining with Ponceau S reagent (Sigma Chemical Co.) and by determining the signal of β-actin.

### Statistical analysis

For each outcome measure, a one-way analysis of variance was performed (SPSS 18.0) for each animal group studied (n = 8). A significant main effect (*p* < 0.05 or *p* < 0.01) was followed-up with Student-Newman-Kuel post hoc comparisons. Values are presented as means ± standard error (SE), and *p* < 0.05 denotes a statistically significant difference.

## Results

### Effect of SNN components on steatosis in HepG2 cells

**Figure 1:**
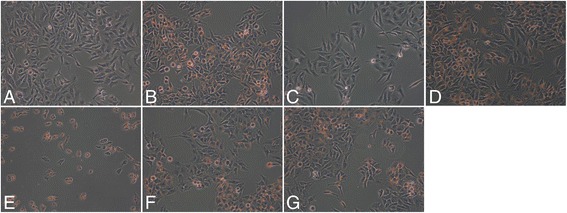
**Effects of SNN chemical components on HepG2 cellular steatosis.** HepG2 cells were stimulated by a mixture of 1 mmol/L FFA (the ratio of oleate to palmitate is 2:1) 24 h. The steatotic cells were then treated with the chemical components for another 24 h. The cells were stained with Oil-Red and observed under microscope (200×). **A**: Control; **B**: Model; **C**: protopanaxadiol 500 μmol/L; **D**: tanshinone IIA 50 μmol/L; **E**: gypenoside 50 μmol/L; **F**: Salvianic acid A sodium 500 μmol/L **G**: Salvianolic acid B 50 μmol/L.

The 24 h incubation HepG2 cells with FFA induced significant lipid accumulation in the cells compared to that of control cells (Figure 1A&B). Treatment with different SNN components to the cell could reduce the lipid to different extent (Figure 1C-G). Notably, protopanaxadiol (500 μmol/L), Salvianic acid A sodium (500 μmol/L), and Salvianolic acid B (50 μmol/L) showed the obvious improvement of the steatosis, indicating the promising effect of SNN components on steatosis in HepG2 cells.

### Parabolic response of serum lipids during hepatosteatosis progressing

**Figure 2:**
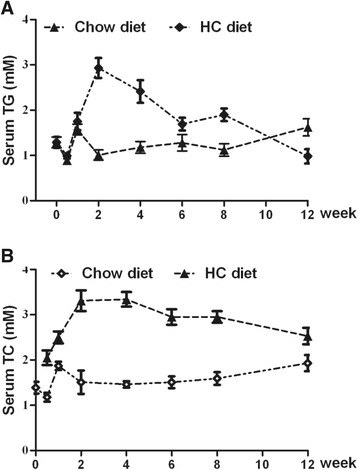
**Parabolic decline of serum triglyceride level upon hepatosteatosis progress.** Male Wistar rats (6 weeks of age) placed on a high-calorie (HC) diet or normal chow diet, blood was collected at 0, 2, 4, 6, 8, 10,12 week, serum was separated and analysed. **A**: The change of serum TG upon HC feeding; **B**: The change of serum TC upon HC feeding.

Male Wistar rats placed on a HC diet exhibited the expected hypertriglyceridemia, hypercholesterolemia, hyperglycemia, hyperinsulinemia, hyperleptinemia, and increased body weight (Table 2) as compared to age matched controls. The serum FFA levels were increased by 30% at the end of 8-week of HC dieting (Table 3); however, the increased fasting FFA concentration upon prolonged HC dieting was not associated with increase in fasting serum TG. Rather, fasting serum TG exhibited gradual decline from 2-week of HC dieting (Figure 2A), even though the hepatic TG content remained high (Table 2). At the end of 12-week of HC dieting, the serum TG concentration was decreased to a level that was lower than that in chow diet-fed animals (chow diet: 1.62 ± 0.80 mM, HC diet: 0.98 ± 0.16 mM; *P* < 0.01). Similarly, serum TC level also showed a trend of decline after the max at 1-week checkpoint, while hepatic cholesterol reached the plateau (Figure 2B). The decrease in plasma TG probably reflects the compromised ability of the liver to produce VLDL after prolonged HC dieting.

**Table 2:** **Liver Parameters**

**Parameters**	**8-week dieting**		**Post 4-week treatment**				
	**Chow**	**HC**	**Chow**	**HC**	**HC + Pio**	**HC + SNN**	**HC + Sim**
**Body weight (g)**	392.6 ± 5.9	433.3 ± 6.1^**††**^	436.0 ± 6.5	487.1 ± 10.7^**^	510.4 ± 11.5	477.3 ± 9.5	477.9 ± 18.9
**Post 4-week body weight gain (g)**	ND	ND	43.38 ± 3.82	53.88 ± 6.81^*^	78.73 ± 8.24^**#**^	45.00 ± 5.96	49.50 ± 7.88
**Liver weight (g)**	9.9 ± 0.07	18.7 ± 0.40^**††**^	14.05 ± 0.39	20.08 ± 0.67^**^	18.55 ± 0.48	19.78 ± 0.70	20.72 ± 1.07
**Liver/Body weight ratio (%)**	2.69 ± 0.02	4.31 ± 0.03	3.15 ± 0.02	4.12 ± 0.17	3.64 ± 0.14	4.14 ± 0.22	4.32 ± 0.22
**Food intake (kal/d)**	73.5 ± 3.1	102.2 ± 3.8^**††**^	75.60 ± 4.0	110.24 ± 5.2^**^	112.36 ± 3.8	106.54 ± 6.1	104.88 ± 5.6
**Liver TG (mg/g)**	20.2 ± 0.6	58.3 ± 1.2^**††**^	24.1 ± 0.6	67.2 ± 2.6^**^	48.1 ± 0.9^**##**^	47.8 ± 1.3^**##**^	46.2 ± 13^**##**^
**Liver glycogen (mg/g)**	ND	ND	46.7 ± 4.57	16.8 ± 1.33^**^	30.8 ± 1.95^**##**^	31.02 ± 1.62^**##**^	ND
**ALT (u/l)**	51.6 ± 2.8	54.4 ± 2.7	56.04 ± 8.91	52.28 ± 5.60	62.51 ± 8.68	70.19 ± 12.76	66.4 ± 8.75
**AST (u/l)**	125.5 ± 12.9	88.8 ± 3.6	110.1 ± 10.79	91.4 ± 12.59	90.2 ± 15.35	132.4 ± 87.98	89.7 ± 8.70

Values are means ± SE (n = 6–8 per group). ^**††**^ *p* < 0.01relative to 8-weekunder chow diet,^*^ *p* < 0.05, ^**^ *p* < 0.01relative to 12-weekunder chow diet; ^**#**^ *p* < 0.05,^**##**^ *p* < 0.01, *versus*12-weekHC diet. *TG*, triglyceride; *ALT*, alanine transaminase; *AST*, aspartate transaminase; *ND*, not determined.

**Table 3:** **Serum Parameters**

**Parameters**	**8-week dieting**		**Post 4-week treatment**				
	**Chow**	**HC**	**Chow**	**HC**	**HC + Pio**	**HC + SNN**	**HC + Sim**
TG (mM)	1.12 ± 0.08	1.90 ± 0.14^**††**^	1.62 ± 0.80	0.98 ± 0.16^**^	0.88 ± 0.23	1.53 ± 0.38^**##**^	1.41 ± 0.22^**##**^
TC (mM)	1.59 ± 0.04	2.95 ± 0.13^**††**^	1.93 ± 0.18	2.53 ± 0.18^**^	2.43 ± 0.13	2.44 ± 0.14	2.46 ± 0.15
HDL-c (mM)	1.02 ± 0.01	0.80 ± 0.04^**††**^	1.66 ± 0.15	0.95 ± 0.09^**^	1.12 ± 0.12^**#**^	0.99 ± 0.06	0.88 ± 0.03
LDL-c (mM)	0.32 ± 0.01	1.60 ± 0.08^**††**^	0.25 ± 0.07	1.47 ± 0.18^**^	1.19 ± 0.16^**#**^	1.17 ± 0.15^**#**^	1.31 ± 0.11
FFA (u/l)	1792 ± 81.6	2414 ± 145^**††**^	1966 ± 107	2562 ± 153^**^	2162 ± 136^**#**^	2100 ± 162^**#**^	2476 ± 174
FBG (mM)	5.0 ± 0.11	8.8 ± 0.26^**††**^	5.5 ± 0.26	9.8 ± 0.26^**^	9.6 ± 0.33	9.7 ± 0.29	ND
FIN (u/l)	36.7 ± 1.27	194.5 ± 25.47^**††**^	56.4 ± 14.6	226.3 ± 19.7^**^	208.0 ± 19.4	219.3 ± 20.2	ND

Values are means ± SE (n =6-8 per group). ^**††**^ *p* < 0.01relative to 8-weekunder chow diet,^**^ *p* < 0.01relative to 12-weekunder chow diet; ^**#**^ *p* < 0.05, ^**##**^ *p* < 0.01, *versus*12-weekHFHS diet.*TG*, triglyceride; *TC*, total cholesterol; *HDL-c*, high density lipoprotein cholesterol; *LDL-c*, low density lipoprotein cholesterol; *FFA*, free fatty acid; *FBG*, fasting blood glucose, *FIN*, fasting insulin; *ND*, not determined.

### Effect of SNN on liver steatosis

**Figure 3:**
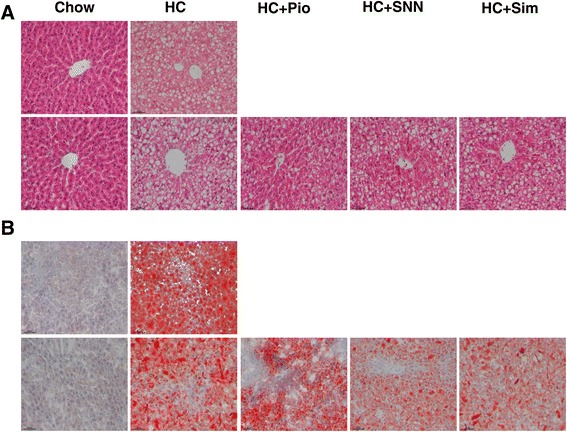
**The effect of SNN on HC diet induced hepatosteatosis.** Male Wistar rats (6 weeks old) were placed on chow diet or HC diet for 8 weeks followed by the 4-week SNN intervention. The livers were excised, processed and stained with hematoxylin-eosin (HE) **(A)** and Oil Red O **(B)** (up*per panels, chow diet; lower panels, HC diet*). Images are magnified 200 × .

The 8-week HC fed rats developed hepatomegaly; the mean liver weight and liver weight/body weight ratio were 2- and 1.6-fold, respectively, higher than that in age matched rats fed chow diet (Table 2). Massive macro- vesicular steatosis were observed in liver sections in accordance with the increase of hepatic TG concentrations, indicating the development of fatty liver in HFHS fed rats. After 4 weeks of SNN treatment, however, hepatic steatosis was markedly reduced, despite the persistence of hepatomegaly (Figure 3). Hepatic TG concentrations in SNN-treated rats were decreased by approximately 30% as compared to that of non-treated control animals, and the effect of SNN treatment in reducing hepatic TG content was comparable to that of Simvastatin and Pioglitazone (Figure 3, Table 2).

### Effect of SNN on serum lipid profile

Analysis of serum lipoproteins revealed that the hypercholesterolemia in rats fed the HC diet was associated with increased LDL-c and decreased HDL-c (Table 3), typical phenotypes of diabetic dyslipidemia. Rats fed with HC diet for 12 weeks also showed increased FFA levels in circulation (Table 3). The 4-week SNN treatment normalized the lipid profile to a certain extent, as manifested by decreased LDL-c and FFA (Table 3).

Increase hepatic fat content often correlates with increased TG secretion in the form of VLDL. However, decrease in serum TG is often observed as fatty liver progresses as a result of impaired hepatocellular function. The decrease in serum TG in HC diet-fed rats was reversed by 4-week SNN treatment (Table 3), suggesting restoration of normal hepatic TG secretion and hepatocellular function.

### Effect of SNN on insulin sensitivity

**Figure 4:**
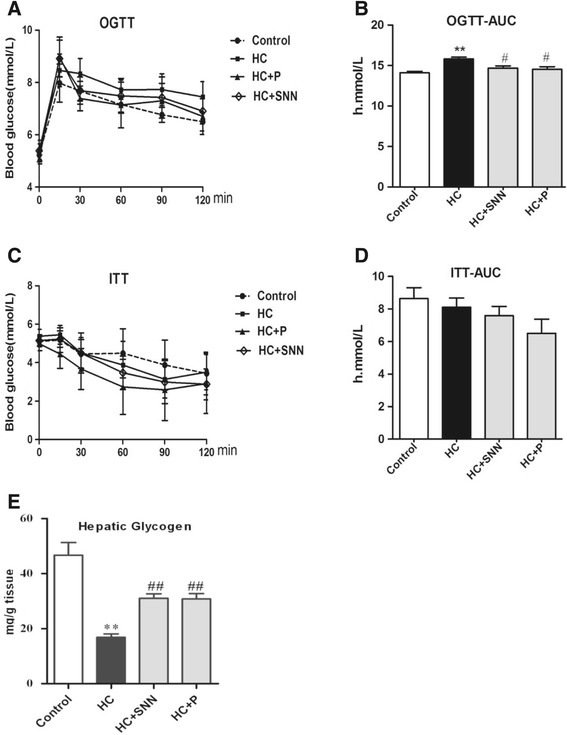
**The effect of SNN on glucose and insulin tolerance and glycogen.** Male Wistar rats (6 weeks old) were placed on chow diet or HC diet for 8 weeks followed by the 4-week SNN intervention. OGTT and ITT were conducted after 6 h fasting, glucose (1.5 g/kg body weight) was orally administered, or insulin (0.75u/kg body weight) was intraperitoneally injected, tail-vein blood samples were collected at baseline and at indicated time intervals (15, 30, 60, 90, and 120 min) after glucose administration. Blood glucose levels were determined with a diabetes monitoring strip. OGTT and ITT curves were drawn **(A, C)** and AUC were calculated **(B, D)**. The livers were excised and glycogen content was detected **(E)**. Data (mean ± SE) are presented, ^**^ *P* < 0.01 relative to control group, ^#^ *P* < 0.05, ^##^ *P* < 0.01 in comparison to HC group.

HC diet-fed rats showed hyperinsulinemia and hyperglycemia, elevated HOMA-IR and impaired glucose tolerance compared with chow diet-fed controls (Table 2), indicative of insulin resistance. With 4-week SNN treatment, decrease in blood glucose levels was observed as compared with that in untreated HC rats at all time points (from 30 min through 2 h) during the OGTT experiment (Figure 4A). Accordingly, decrease in OGTT-AUC values ensured in the SNN-treated rats (Figure 4B). As for ITT, there is no significant difference of the blood glucose between control group and HC diet group (Figure 4C), and so did the ITT-AUC values (Figure 4D). The 12-week HC feeding also resulted in a 60% decrease of hepatic glycogen content as compared to chow diet-fed rats, and SNN treatment partially normalized the hepatic glycogen content (Figure 4E), and the effect of SNN was comparable to that of pioglitazone.

**Figure 5:**
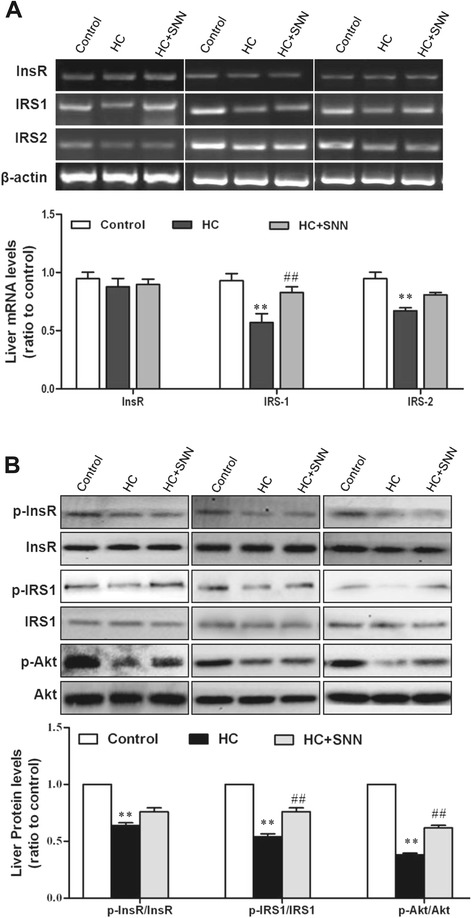
**The effect of SNN on InsR, IRS1/2 and Akt expression in the liver.** Male Wistar rats (6 weeks old) were placed on chow diet or HC diet for 8 weeks followed by the 4-week SNN intervention. Livers were collected, the mRNA expression of InsR and IRS1/2 was detected through PCR **(A)**, and the protein expression of Total and phosphorylated InsR (Tyr^1131^), IRS1 (Ser^302^), Akt (Ser^473^) was analysed by Western blots **(B)**. Data (mean ± SE) are presented as relative expression levels compared to that of control group. ^**^ *P* < 0.01 relative to control group, ^##^ *P* < 0.01 in comparison to HC group.

In comparison to that in rats fed chow diet, the levels of InsR and IRS mRNA and IRS1 phosphorylation were decreased in rats fed HC diet (Figure 5). To demonstrate the effect of SNN in regulating insulin signalling transduction, we analysed molecular expression in insulin signalling pathway. With SNN supplement, the phosphorylated InsR and IRS2 levels were not significantly different from that of untreated rats, but IRS1 mRNA expression (Figure 5A) and phosphorylation protein (Figure 5B) dramatically increased, indicating the restoration of IRS1 activity. Akt is one of the downstream molecules of IRS in insulin pathway, which is critical in glucose homeostasis because it controls both gluconeogenesis and glycogen synthesis. The Akt phosphorylation in the 12-week HC rats were remarkably decreased, which may partially explain the 60% decrease of hepatic glycogen content compared with the chow diet animals. SNN increased the hepatic Akt phosphorylation (Figure 5A) and glycogen content (Figure 5B) with the 4-week treatment. These data together suggest strongly an improvement in hepatic insulin sensitivity with SNN treatment.

### Effect of SNN on regulatory molecules of insulin pathway

**Figure 6:**
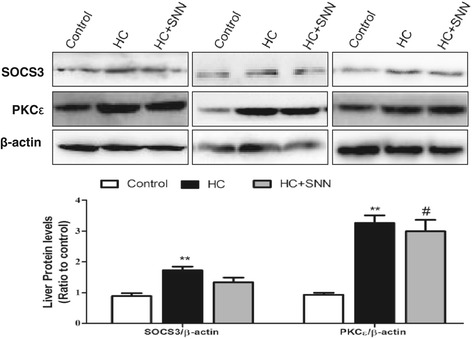
**The effect of SNN on SOCS3 and PKC** **ε** **expression in the liver.** Male Wistar rats (6 weeks old) were placed on chow diet or HC diet for 8 weeks followed by the 4-week SNN intervention. Livers were collected, the protein expression of SOCS3 and PKCε was analysed by Western blots. Data (mean ± SE) are presented as relative expression levels compared to that of control group. ^**^ *P* < 0.01 relative to control group, ^#^ *P* < 0.05 in comparison to HC group.

The abnormalities in lipid and glucose metabolism upon HC dieting were also associated with activation of regulatory signaling pathways. HC dieting induced increased expression of SOCS3, a negative regulator insulin signaling. SOCS3 has been shown interfering insulin signaling by competitively binding to the InsR, affecting IRS phosphorylation [17], or promoting ubiquitin-mediated IRS degradation [18]. Indeed, upon HC dieting, SOCS3 expression in the liver was up-regulated upon 12-week HC dieting, and with 4-week SNN treatment, SOCS3 expression was significantly decreased (Figure 6), indicating SNN has an inhibitory effect on SOCS3 expression, and the change was also consistent with IRS expression. In addition to SOCS3, the lipid-dependent kinases, PKC family has been reported to inhibit several components of the insulin signaling pathway, among the 10 isoforms, PKCε is the isoform most often implicated in association with hepatic insulin resistance [19]. Western blot analysis revealed that PKCε was markedly increased along with the development of hepatic steatosis, however, the regulation of SNN on PKCε was statistically no significant (Figure 6).

## Discussion

Natural plant products provide a number of promising therapeutic agents. TCM uses a large variety of such herbal products and provides a rational prescription approach that targets the pattern of the syndrome, which is the essence of its therapeutic strategy. A lipid-lowering agent SNN was designed based on the characteristics of NAFLD and on TCM theory. Clinical studies confirmed SNN as a therapeutic option for the development and progression of NAFLD [20]. The current study has demonstrated the SNN chemical components can reduce FFA-induced cellular steatosis. While HC diet-induced NAFLD rats exhibited dyslipidemia, hyperinsulinemia, and insulin resistance with concomitant hepatic steatosis, SNN reduced serum levels of LDL-c, and hepatic levels of TG with an attenuation of hepatic steatosis in HC diet-induced rats. These results together indicate that SNN is an effective drug for treating NAFLD.

Hepatic steatosis is the hallmark of NAFLD, and TG accumulation in hepatocytes is considered to be the major pathogenic trigger in the development of NAFLD [21]. In the current study, SNN components could improve FFA-induced steatosis in HepG2 cells, and animal studies showed that SNN could significantly attenuate the HC diet-induced hepatic steatosis and the lipid accumulation, both of which confirmed the therapeutic effect of SNN. Liver also overproduces several atherogenic factors under nutritional stress [22,23], in this study, the levels of and LDL-c were significantly decreased in SNN-treated rats, which partly confirms the protective nature of SNN against atherogenic risk.

NAFLD is associated with dyslipidemia, increased hepatic fat content often (but not always) correlates with increased hepatic TG secretion. The only route for TG export from hepatocytes is via the assembly of VLDL. However, as NAFLD progresses, the production of VLDL might decrease secondary to the impairment of hepatocellular function. Studies of seven patients with biopsy proven NASH showed decreased apolipoprotein B (apoB, the primary apolipoproteins of VLDL) production rate (by 50%) as compared with obese or lean controls without NAFLD [24]. We have observed a parabolic response of serum TG level during diet-induced fatty liver progression, indicating the impairment of hepatocellular function. The mechanisms responsible for the parabolic change of serum TG is unclear. Nevertheless, 4-week SNN treatment results in an improved hepatocellular function, evidenced by the restoration of serum TG. Cell culture and animal studies showed that, although moderate FFA overload increases VLDL secretion, prolonged FFA exposure induced ER stress and resulted in decreased VLDL secretion [25]. Whether or not SNN treatment can reduce ER stress remains to be determined.

The two-hit and multi-hit hypotheses postulate that insulin resistance is the initial trigger of the pathogenesis of NAFLD [26,27]. Insulin sensitivity/resistance is usually tested on glucose metabolism. HOMA-IR is largely used in epidemiological studies because it only requires measuring FIN and FBG, and OGTT indices are frequently used in clinical practice [28,29]. Both tests are commonly used to measure whole-body and peripheral insulin resistance. The results of the experiment in this study showed an increase of HOMA-IR and OGTT indices, which signify insulin insensitivity in NAFLD rats. SNN markedly reduces OGTT indices, but fails to inhibit HOMA-IR increase, which suggests that SNN may not completely reverse whole-body insulin resistance involved in NAFLD.

Insulin has a strong influence on lipogenesis and glucose homeostasis as well [30,31]. The failure of hepatocytes to respond to insulin contributes to the development of glucose intolerance, whereas lipogenesis, which is positively regulated by insulin, remains sensitive to insulin and is driven excessively by the compensatory hyperinsulinemia [32]. Thus, the failure of the insulin system enhances hepatic glucose and lipid production [33]. Hepatic insulin resistance is strongly associated with NAFLD and is characterized by the impairment of glycogenesis with an increase of glucogenesis and glycogenolysis [34]. Glycogen synthesis represents a major pathway for glucose disposal after insulin stimulation, and increased hepatic glycogen synthesis and glycogen content are involved in enhanced insulin sensitivity [35]. Previous studies suggested that glycogen in the liver is important to maintain physical performance during prolonged exercise, and that glycogen synthesis is reduced in type 2 diabetes mellitus [36,37]. The present study demonstrated that SNN increases hepatic glycogen content, which is parallel to its improvement of insulin signalling transduction in NAFLD rats. The decrease in liver InsR and IRS expression is in accordance with the impairment of the hepatic insulin pathway and represents the therapeutic targets of insulin resistance [16]. This finding indicates that the improvement of hepatic insulin sensitivity contributes to the reduction of hepatic lipid accumulation in NAFLD rats. Therefore, hepatic insulin resistance is an important target for SNN therapy for HC diet-induced NAFLD.

Hepatic insulin resistance is also associated with activation of regulatory signaling pathways. Among the regulatory molecules, SOCS3 has been shown interfering insulin signaling by competitively binding to the InsR, affecting IRS phosphorylation [17], or promoting ubiquitin-mediated IRS degradation [18]. In addition to SOCS3, the isoforms of PKC family have wide-ranging roles in signal transduction, including the positive and negative modulation of insulin action, and they are dependent on diacylglycerol (DAG) for full activation [38]. PKCε is the isoform most often implicated in association with hepatic insulin resistance, PKCε in liver may affect its ability to phosphorylate the substrates or InsR trafficking in hepatocytes [39]. In our study, both SOCS3 and PKCε were significantly increased with the development of NAFLD, which was consistent with the impairment of insulin signaling pathway, indicating these regulatory molecules may contribute to the insulin resistance. SNN showed improvement on SOCS3 expression, but not that of PKCε, the potential mechanisms need to be further explored, other isoforms of PKC family may also involved in the regulatory process.

Thiazolidinediones are insulin-sensitizing agents that improve insulin sensitivity and are effective for the treatment of specific metabolic defects. However, the use of thiazolidinediones for NAFLD treatment raises a number of safety concerns [40,41]. In this study, pioglitazone shows beneficial effects on NAFLD rats, but causes body weight gain, which is in accordance with the results in reported clinical studies [42,43]. Moreover, pioglitazone may predispose at-risk patients to exacerbated heart failure [44]. Because of these side effects in addition to other potential risks, efficacy of some of the insulin sensitizers in NAFLD therapy is limited. This study shows that the two regimens for treating NAFLD rats have similar effects. The results also show that SNN rats do not exhibit any body weight gain, which signifies that SNN is safe and suitable for long-term use.

## Conclusions

The results show that SNN exerts a protective effect on steatosis in HepG2 cells and HC diet-induced NAFLD in rats. This protective effect may be due to attenuation of hepatic insulin resistance.

## References

[1] Adams LA, Waters OR, Knuiman MW, Elliott RR, Olynyk JK (2009). NAFLD as a risk factor for the development of diabetes and the metabolic syndrome: an eleven-year follow-up study. Am J Gastroenterol.

[2] Janczyk W, Socha P (2012). Non-alcoholic fatty liver disease in children. Clin Res Hepatol Gastroenterol.

[3] Abdelmalek MF, Diehl AM (2007). Nonalcoholic fatty liver disease as a complication of insulin resistance. Med Clin North Am.

[4] Marchesini G, Moscatiello S, Di Domizio S, Forlani G (2008). Obesity-associated liver disease. J Clin Endocrinol Metab.

[5] Utzschneider KM, Kahn SE (2006). Review: The role of insulin resistance in nonalcoholic fatty liver disease. J Clin Endocrinol Metab.

[6] Previs SF, Withers DJ, Ren JM, White MF, Shulman GI (2000). Contrasting effects of IRS-1 versus IRS-2 gene disruption on carbohydrate and lipid metabolism in vivo. J Biol Chem.

[7] Bowman TA, Ramakrishnan SK, Kaw M, Lee SJ, Patel PR, Golla VK, Bourey RE, Haram PM, Koch LG, Britton SL, Wisloff U, Lee AD, Najjar SM (2010). Caloric restriction reverses hepatic insulin resistance and steatosis in rats with low aerobic capacity. Endocrinology.

[8] Wang M, Sun S, Wu T, Zhang L, Song H, Hao W, Zheng P, Xing L, Ji G (2013). Inhibition of LXRalpha/SREBP-1c-Mediated Hepatic Steatosis by Jiang-Zhi Granule. Evid Based Complement Alternat Med.

[9] LU Y-L WANGM, ZHANG L, HE Y-Q YANGL, WANG C-H, WANG Z-T JIG (2010). Simultaneous Determination of Six Components in the ‘Jiang-Zhi’ Granule by UPLC-MS Analysis. Chin J Nat Med.

[10] Qi Z, Xue J, Zhang Y, Wang H, Xie M (2011). Osthole ameliorates insulin resistance by increment of adiponectin release in high-fat and high-sucrose-induced Fatty liver rats. Planta Med.

[11] Lomba A, Milagro FI, Garcia-Diaz DF, Marti A, Campion J, Martinez JA (2010). Obesity induced by a pair-fed high fat sucrose diet: methylation and expression pattern of genes related to energy homeostasis. Lipids Health Dis.

[12] Fernandes-Santos C, Evangelista Carneiro R, De Souza ML, Barbosa Aguila M, Mandarim-de-Lacerda CA (2009). Rosiglitazone aggravates nonalcoholic Fatty pancreatic disease in C57BL/6 mice fed high-fat and high-sucrose diet. Pancreas.

[13] Song HY, Zhang L, Pan JL, Yang LL, Ji G (2013). Bioactivity of five components of Chinese herbal formula Jiangzhi granules against hepatocellular steatosis. J Integr Med.

[14] Lima CR, Vasconcelos CF, Costa-Silva JH, Maranhao CA, Costa J, Batista TM, Carneiro EM, Soares LA, Ferreira F, Wanderley AG (2012). Anti-diabetic activity of extract from Persea americana Mill. leaf via the activation of protein kinase B (PKB/Akt) in streptozotocin-induced diabetic rats. J Ethnopharmacol.

[15] Villanueva CJ, Monetti M, Shih M, Zhou P, Watkins SM, Bhanot S, Farese RV (2009). Specific role for acyl CoA:Diacylglycerol acyltransferase 1 (Dgat1) in hepatic steatosis due to exogenous fatty acids. Hepatology.

[16] Xing LJ, Zhang L, Liu T, Hua YQ, Zheng PY, Ji G (2011). Berberine reducing insulin resistance by up-regulating IRS-2 mRNA expression in nonalcoholic fatty liver disease (NAFLD) rat liver. Eur J Pharmacol.

[17] Ueki K, Kondo T, Kahn CR (2004). Suppressor of cytokine signaling 1 (SOCS-1) and SOCS-3 cause insulin resistance through inhibition of tyrosine phosphorylation of insulin receptor substrate proteins by discrete mechanisms. Mol Cell Biol.

[18] Rui L, Yuan M, Frantz D, Shoelson S, White MF (2002). SOCS-1 and SOCS-3 block insulin signaling by ubiquitin-mediated degradation of IRS1 and IRS2. J Biol Chem.

[19] Considine RV, Nyce MR, Allen LE, Morales LM, Triester S, Serrano J, Colberg J, Lanza-Jacoby S, Caro JF (1995). Protein kinase C is increased in the liver of humans and rats with non-insulin-dependent diabetes mellitus: an alteration not due to hyperglycemia. J Clin Invest.

[20] Pan J, Wang M, Song H, Wang L, Ji G (2013). The efficacy and safety of traditional chinese medicine (jiang zhi granule) for nonalcoholic Fatty liver: a multicenter, randomized, placebo-controlled study. Evid Based Complement Alternat Med.

[21] Fon Tacer K, Rozman D (2011). Nonalcoholic Fatty liver disease: focus on lipoprotein and lipid deregulation. J Lipids.

[22] Musunuru K (2010). Atherogenic dyslipidemia: cardiovascular risk and dietary intervention. Lipids.

[23] Poss J, Custodis F, Werner C, Weingartner O, Bohm M, Laufs U (2011). Cardiovascular disease and dyslipidemia: beyond LDL. Curr Pharm Des.

[24] Charlton M, Sreekumar R, Rasmussen D, Lindor K, Nair KS (2002). Apolipoprotein synthesis in nonalcoholic steatohepatitis. Hepatology.

[25] Ota T, Gayet C, Ginsberg HN (2008). Inhibition of apolipoprotein B100 secretion by lipid-induced hepatic endoplasmic reticulum stress in rodents. J Clin Invest.

[26] Day CP, James OF (1998). Steatohepatitis: a tale of two “hits”?. Gastroenterology.

[27] Jou J, Choi SS, Diehl AM (2008). Mechanisms of disease progression in nonalcoholic fatty liver disease. Semin Liver Dis.

[28] Gayoso-Diz P, Otero-Gonzalez A, Rodriguez-Alvarez MX, Gude F, Cadarso-Suarez C, Garcia F, De Francisco A (2011). Insulin resistance index (HOMA-IR) levels in a general adult population: curves percentile by gender and age. The EPIRCE study. Diabetes Res Clin Pract.

[29] Mari A, Pacini G, Murphy E, Ludvik B, Nolan JJ (2001). A model-based method for assessing insulin sensitivity from the oral glucose tolerance test. Diabetes Care.

[30] Ben Djoudi Ouadda A, Levy E, Ziv E, Lalonde G, Sane AT, Delvin E, Elchebly M (2009). Increased hepatic lipogenesis in insulin resistance and Type 2 diabetes is associated with AMPK signalling pathway up-regulation in Psammomys obesus. Biosci Rep.

[31] Zheng D, Ionut V, Mooradian V, Stefanovski D, Bergman RN (2009). Exenatide sensitizes insulin-mediated whole-body glucose disposal and promotes uptake of exogenous glucose by the liver. Diabetes.

[32] Biddinger SB, Hernandez-Ono A, Rask-Madsen C, Haas JT, Aleman JO, Suzuki R, Scapa EF, Agarwal C, Carey MC, Stephanopoulos G, Cohen DE, King GL, Ginsberg HN, Kahn CR (2008). Hepatic insulin resistance is sufficient to produce dyslipidemia and susceptibility to atherosclerosis. Cell Metab.

[33] Farese RV, Zechner R, Newgard CB, Walther TC (2012). The Problem of Establishing Relationships between Hepatic Steatosis and Hepatic Insulin Resistance. Cell Metab.

[34] Bugianesi E, McCullough AJ, Marchesini G (2005). Insulin resistance: a metabolic pathway to chronic liver disease. Hepatology.

[35] Zhang Y, Lee FY, Barrera G, Lee H, Vales C, Gonzalez FJ, Willson TM, Edwards PA (2006). Activation of the nuclear receptor FXR improves hyperglycemia and hyperlipidemia in diabetic mice. Proc Natl Acad Sci U S A.

[36] Ros S, Garcia-Rocha M, Calbo J, Guinovart JJ (2011). Restoration of hepatic glycogen deposition reduces hyperglycaemia, hyperphagia and gluconeogenic enzymes in a streptozotocin-induced model of diabetes in rats. Diabetologia.

[37] Ha Do T, Trung TN, Hien TT, Dao TT, Yim N, Ngoc TM, Oh WK, Bae K (2010). Selected compounds derived from Moutan Cortex stimulated glucose uptake and glycogen synthesis via AMPK activation in human HepG2 cells. J Ethnopharmacol.

[38] Schmitz-Peiffer C, Biden TJ (2008). Protein kinase C function in muscle, liver, and beta-cells and its therapeutic implications for type 2 diabetes. Diabetes.

[39] Hribal ML, D’Alfonso R, Giovannone B, Lauro D, Liu YY, Borboni P, Federici M, Lauro R, Sesti G (2001). The sulfonylurea glimepiride regulates intracellular routing of the insulin-receptor complexes through their interaction with specific protein kinase C isoforms. Mol Pharmacol.

[40] McCowen KC, Fajtova VT (2011). Pioglitazone for diabetes prevention. N Engl J Med.

[41] Aithal GP, Thomas JA, Kaye PV, Lawson A, Ryder SD, Spendlove I, Austin AS, Freeman JG, Morgan L, Webber J (2008). Randomized, placebo-controlled trial of pioglitazone in nondiabetic subjects with nonalcoholic steatohepatitis. Gastroenterology.

[42] Kusunoki M, Tsutsumi K, Sato D, Nakamura A, Habu S, Mori Y, Morishita M, Yonemoto T, Miyata T, Nakaya Y, Nakamura T (2011). Pioglitazone-induced body weight gain is prevented by combined administration with the lipoprotein lipase activator NO-1886. Eur J Pharmacol.

[43] Sanyal AJ, Chalasani N, Kowdley KV, McCullough A, Diehl AM, Bass NM, Neuschwander-Tetri BA, Lavine JE, Tonascia J, Unalp A, Van Natta M, Clark J, Brunt EM, Kleiner DE, Hoofnagle JH, Robuck PR, Nash CRN (2010). Pioglitazone, vitamin E, or placebo for nonalcoholic steatohepatitis. N Engl J Med.

[44] Lincoff AM, Wolski K, Nicholls SJ, Nissen SE (2007). Pioglitazone and risk of cardiovascular events in patients with type 2 diabetes mellitus: a meta-analysis of randomized trials. JAMA.

